# A Rare Case of a Metastatic Lung Squamous Cell Carcinoma to the Large Bowel and the Liver

**DOI:** 10.7759/cureus.13867

**Published:** 2021-03-13

**Authors:** Nikolaos Pararas, Georgios Kirkilessis, Andreas Pikoulis, Konstantinos Syrigos, Emmanouil Pikoulis

**Affiliations:** 1 General Surgery, Dr Sulaiman Al Habib/Alfaisal University, Riyadh, SAU; 2 Third Department of Surgery, Attikon University Hospital, National and Kapodistrian University of Athens, Athens, GRC; 3 Oncology Unit, Third Department of Medicine, "Sotiria" Hospital for Diseases of the Chest, National and Kapodistrian University of Athens, Athens, GRC

**Keywords:** lung cancer, squamous cell carcinoma, metastasis to the colon

## Abstract

Lung carcinoma is the leading cause of death worldwide, with almost 50% of the patients presenting with distant metastases at the moment of diagnosis. The most common metastases sites are the lymph nodes, the liver, the adrenal glands, the bones, and the brain. The gastrointestinal tract was considered an unusual site for lung metastases, and due to the asymptomatic progress of the disease, they are usually diagnosed at a late stage. In the present case study, the clinical presentation, the treatment, and outcome of a rare case of descending colon and liver metastases from a lung squamous cell carcinoma in a 72-year-old female, two years after the presentation of her primary tumor are reported. The present study aims to increase the awareness for early diagnosis and treatment of metastatic lung cancer to the gastrointestinal tract.

## Introduction

Lung carcinoma is one of the most diagnosed cancers globally and is the leading cause of death in men and the second cause of death in women worldwide [1]. Almost 50% of all lung cancers have distal metastases at the time of diagnosis, with the most frequent metastasis sites being the brain, the liver, the bones, and the adrenal glands [2,3].

Autopsy studies reported that gastrointestinal metastases from primary lung cancer occur in 0.2%-14% of the cases [2,4-8]. Analysis of these studies reveals that the rate of gastrointestinal metastases from primary lung cancer is more frequent than initially believed. On the contrary, symptomatic gastrointestinal metastasis from the lungs’ clinical prevalence is only 0.2%-0.5%. The most common metastatic site is the small bowel, with sporadic case reports of the stomach, large bowel, and anus being reported [5,9-12]. Due to this asymptomatic progression, most gastrointestinal metastases from the lung cases are diagnosed at a late stage with a poor prognosis [13], and the management of such cases remains controversial [14,15].

In the present case study, the clinical presentation, the treatment, and outcome of a rare case of descending colon and liver metastases from a lung squamous cell carcinoma are reported. The present study may increase the awareness for early diagnosis and treatment of metastatic lung cancer to the gastrointestinal tract.

## Case presentation

The patient was a 72-year-old female, heavy smoker (60 pack/years) with an extensive medical history, including obesity (weight 70 kg, height 1.50 m, BMI 31.1), hypertension, type II diabetes, hyperlipidemia, and depression, under treatment, and free family history. The patient initially presented to her primary care doctor in August 2018, suffering from blood spots in her sputum that were present for 15 days before her visit. CT of the chest revealed a space-occupying lesion of the upper right lobe, measuring 2.4x3 cm, compatible with lung carcinoma. Further preoperative staging with positron emission tomography (PET) showed no other synchronous primary tumors, secondary depositions, or metastases elsewhere. A right upper lobectomy was performed in October 2018, and histological examination of the resected specimen revealed a 3.4x2.5x3.1 cm squamous cell carcinoma of low differentiation, and the immunohistochemistry showed CK19 (+), S-Bpr(-), TTF-1(-), SMA (+) in the Ki-67(+) fibroblastic substrate. According to the tumor nodes metastases (TNM) classification, the tumor was pT2N0M0.

Postoperatively, she received first-line adjuvant chemotherapy with carboplatin/paclitaxel (2/2019-5/2019) and radiation therapy (until 8/2019), and since then, she had been on a follow up with the Oncology Department. In August 2020, upon her regular follow-up, the CT abdomen showed thickening of the wall of the descending colon and a single hepatic lesion at segment 8 of the liver, which was verified by a liver MRI and measured 17x11mm and was characterized as a metastasis. A lesion on the right adrenal gland was evident as well, which was characterized as an adenoma and was present in previous imaging, unchanged in size. Her clinical examination was unremarkable. No masses were palpated in the abdomen, and she had no signs of intestinal obstruction.

**Figure 1:**
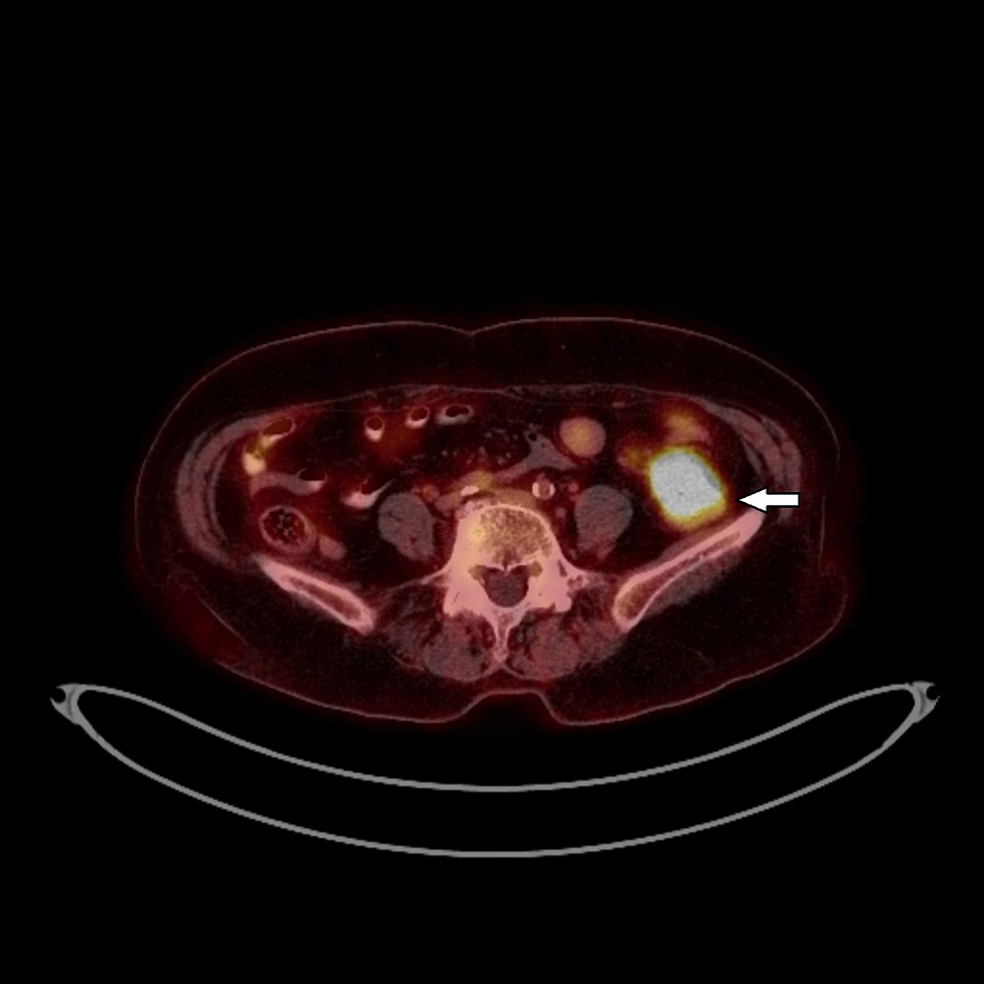
Pre-operative PET-CT showing the descending colon metastasis (arrow) from the squamous cell lung carcinoma. PET-CT: positron emission tomography-computed tomography.

**Figure 2:**
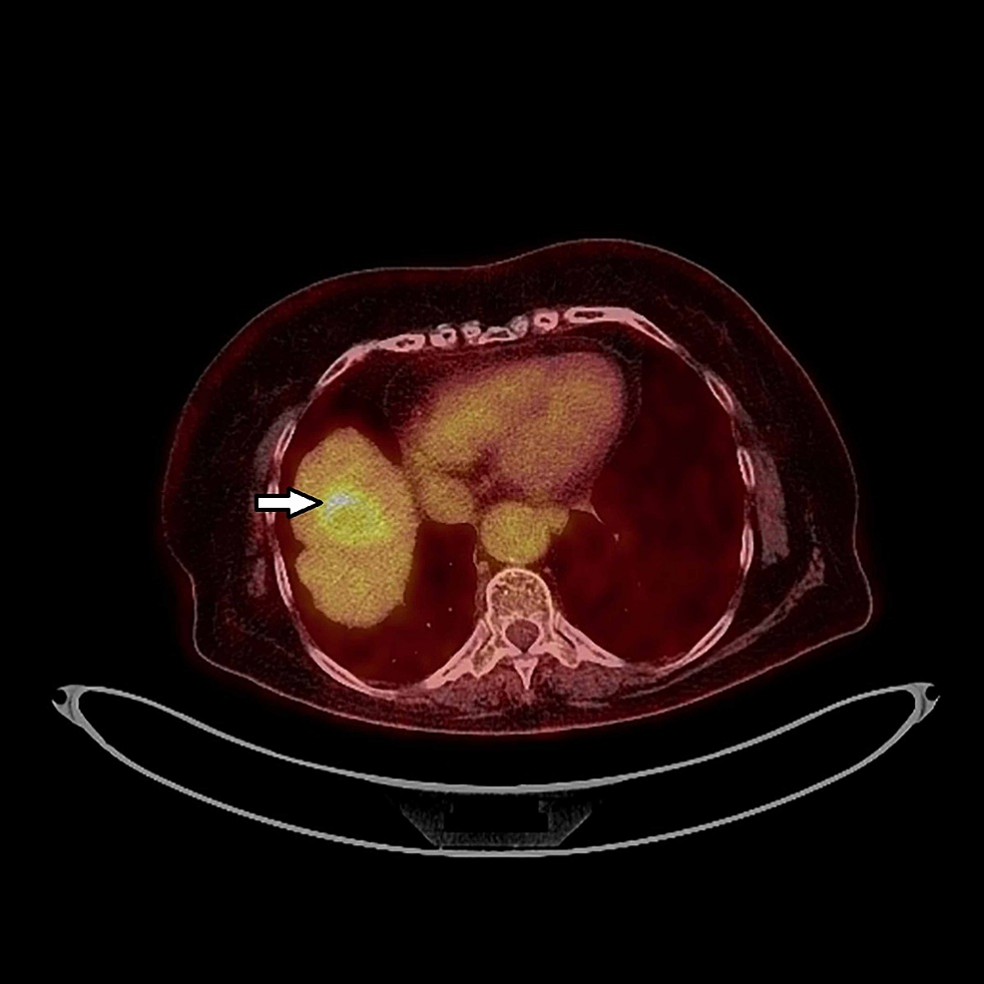
Pre-operative PET-CT showing the single liver metastasis (arrow) from the lung squamous cell carcinoma, in segment VIII. PET-CT: positron emission tomography-computed tomography.

Further evaluation was performed with lower gastrointestinal tract endoscopy, which revealed an almost obstructing tumor of the descending colon, from which biopsies were taken, and the histological examination showed squamous cell carcinoma. In contrast, the immunohistochemistry showed CK 5/6 (+), CK 7 (+), CK 20 (-) CEA (-) and P40 (+). A fine needle biopsy (FNB) was performed for the liver lesion, with the histological examination confirming cancerous cells' presence but not identifying the origin. Finally, the evaluation was concluded with a new PET-CT, which flagged the descending colon lesion [standardized uptake value (SUV)max 24.1] (Figure 1), and the hepatic lesion (SUVmax 5.6) (Figure 2) and did not reveal any additional pathologies. Upon assessment by the multidisciplinary oncology meeting, the colon and the liver lesions were considered metastatic from the lung's primary squamous cell carcinoma, and aggressive surgical resection was advised for the tumors mentioned above as excision of the right adrenal mass. The operation took place on October 2020, after endocrinology evaluation for the adrenal gland tumor, and an open left colectomy with segmentectomy of segment VIII of the liver and excision of the right adrenal gland was performed. An intraoperative ultrasonogram of the liver was performed that revealed the lesion was 5 cm in maximum diameter. Radiofrequency ablation was performed along with segmentectomy of segment VIII. The pathology report showed that the descending colon lesion was a squamous cell carcinoma, invading the serosa, and immunohistochemistry showed CK 5/6 +, p63 (+), CK 7 (+), CK 20 (-), TIFF (-), GATA 3 (-). There were three lymph nodes positive for tumor invasion out of the 13 harvested. The liver lesion was measuring 5.6 cm macroscopically and had the same tumor characteristics as the colon cancer. The surgical margins of all specimens were free of cancer. Finally, the adrenal gland tumor did not have any cancer infiltration.

Postoperatively, the patient was put on steroid supplementation for the adrenalectomy, which was tapered off until the 7th postoperative day, when discontinued. On the fourth day postoperatively, the patient developed sudden dyspnea and orthopnea with chest pain. Pulmonary embolism of the left upper lobe and lower peripheral branches were revealed on CT with pulmonary artery protocol. Cardiology evaluation with laboratory tests and Doppler investigation showed a myocardial infarction and early signs of heart failure. The patient received enoxaparine 80 mg twice daily for the pulmonary embolism and the indicated medical treatment for her heart failure. She did not suffer any other complications and was discharged in good general condition on the 13th postoperative day. On the first follow-up appointment, one month after the operation, the patient was clinically well and is currently followed by the Clinical Oncology Team to continue adjuvant therapy and close follow-up with scheduled short interval CT scans.

## Discussion

Lung carcinoma is the leading cause of death worldwide, with almost 50% of the patients presenting with distant metastases at the moment of diagnosis [1-3]. The most common metastases sites are the lymph nodes, the liver, the adrenal glands, the bones, and the brain [2,3]. Before the 1980s, the gastrointestinal tract was considered an unusual site for lung metastasis [16,17]. Antler et al. [8], in an autopsy review study, reported the incidence to be 14%. Nevertheless, the true incidence must be lower, as a big number of metastatic diseases to the gastrointestinal tract from the lung was by direct tissue invasion to the esophagus. In contrast to the small bowel, which is the most common site of metastases of the gastrointestinal tract from the lung, there have been few reports of lung cancer metastases to the colon [18].

The clinical course is usually insidious, making the diagnosis of such cases difficult, especially in the early stages. In the current study, the patient was diagnosed with a metastatic cancer of the descending colon and a synchronous liver metastasis of squamous cell lung cancer, which other studies have not reported. Symptoms from metastatic gastrointestinal tumors from the lung are rare, and most of them are not noticeable, including abdominal pain, constipation, meteorism, and weight loss [19,20], so the clinical diagnosis of such patients is challenging. Our patient was asymptomatic, and CT scans followed by PET-CT found the metastatic tumors, and the diagnosis was confirmed by colonoscopy and histopathology. CT scan is one of the most offered modalities to the physician to diagnose lung metastases. Gastrointestinal metastases from the lung should be considered in the differential diagnosis when CT scans detect bowel wall thickening in combination with lymphadenopathy [10]. Additionally, PET-CT has good sensitivity and specificity to detect metastatic tumors, and it is recommended in hospitals available [20]. Finally, gastrointestinal endoscopy and histopathologic diagnosis are the golden standards for confirming gastrointestinal tract metastasis from the lung [8].

The histological type of lung cancer associated with gastrointestinal metastases remains incompletely understood. The histologic type of the metastatic gastrointestinal and liver tumors in our patient was squamous cell carcinoma. Some clinical studies have shown that squamous cell carcinoma, large cell carcinoma, and pleomorphic carcinoma of the lung give metastases to the gastrointestinal tract more frequently [17,18]. In contrast, other studies and autopsy reports find adenocarcinoma to be the most typical lung cancer type to metastasize to the gastrointestinal tract [20].

Treatment for these patients is complicated and demands collaboration and coordination of doctors of multiple specialties. It also requires active involvement from the patient in informed decision making, as the course of the disease is unpredictable and will need further adjuvant therapy, which might involve further surgeries and a close follow-up with repeated CT scans. 

Most lung cancer patients with colon metastases will present with bowel perforation or acute abdomen and require a surgical intervention. Postoperative chemotherapy and individualized treatment may improve the survival rate for these patients. Some studies showed that there was no difference in the recurrence-free survival between the patients that received adjuvant chemotherapy and those who received adjuvant chemo-radiotherapy [20].

In this case, our patient was firstly diagnosed with an early-stage I squamous cell carcinoma of the lung two years ago, which was successfully resected, and she received first-line chemotherapy and radiation therapy. Now she presented with a stage IV squamous cell carcinoma of the colon and liver metastasis. Although she had a gastrointestinal metastatic tumor and a liver metastasis, we aggressively treated her, with curative intent, by resecting the colon cancer and the liver metastasis simultaneously. According to Yang et al. [2], from the date of the diagnosis of gastrointestinal metastasis to death was 130 days, indicating a poor prognosis. However, one report showed the survival of one patient five years after resectioning the metastatic colon [5]. There are no well-established evidence-based guidelines for these cases. Furthermore, deciding if and how to treat these patients should be based on a multidisciplinary cancer committee and should be individualized.

Overall, according to the literature, the incidence of gastrointestinal tract metastases from the lung in the same patient is increasing due to the increased early detection and advances in cancer treatment. The treatment of these patients remains challenging because of the lack of well-established treatment guidelines, and their management should be individualized through a multidisciplinary approach. Previous studies have shown that the time interval between the diagnosis of primary lung cancer and the diagnosis of gastrointestinal metastases is ranged between two weeks and four years. Therefore, this should always be kept in mind when treating cancer patients, even though the five-year cure protocol may be valid for the first cancer in most cases. The early detection of a gastrointestinal metastasis will allow prompt management. In the authors’ opinion, an aggressive approach conserving as much as organ function as possible still offers the patients the best chance for long-term survival.

## Conclusions

In conclusion, the colon and liver's synchronous metastases from lung cancer are extremely rare, and the majority of cases is diagnosed at a late stage. Despite newer methodology in diagnosing and treating this condition, the prognosis remains poor. Therefore, the presence of gastrointestinal metastases may be life-threatening and comprehensive evaluations are required to detect and monitor gastrointestinal metastases during follow-up. Finally, new individualized treatment protocols should be made tailored to each case separately. This case study serves as another piece of data in the literature to clearly understand and manage patients with gastrointestinal metastases from the lung. Furthermore, research is needed to clarify the pathogenesis and improve treatment of such patients and the impact that prior therapies have on prognosis and anti-cancer efficacy.
